# The Burden of Undiagnosed Adults With Attention-Deficit/Hyperactivity Disorder Symptoms in Japan: A Cross-Sectional Study

**DOI:** 10.7759/cureus.19615

**Published:** 2021-11-15

**Authors:** Noriyuki Naya, Toshinaga Tsuji, Nobuhiro Nishigaki, Chika Sakai, Yirong Chen, Sungeun Jung, Hirotaka Kosaka

**Affiliations:** 1 Medical Affairs Department, Shionogi & Co. Ltd., Osaka, JPN; 2 Japan Medical Office, Takeda Pharmaceutical Company Limited, Tokyo, JPN; 3 Health Division, Kantar, Singapore, SGP; 4 Health Division, Kantar, Seoul, KOR; 5 Department of Neuropsychiatry, Faculty of Medical Sciences, University of Fukui, Fukui, JPN

**Keywords:** undiagnosed adhd, resource utilization, work productivity, quality of life, adhd symptoms with comorbidities, asrs

## Abstract

Background

Symptoms experienced by adult patients with attention-deficit/hyperactivity disorder (ADHD) frequently result in functional impairment across academic/occupational functioning, daily life, and social functioning. A substantial proportion of undiagnosed and untreated ADHD has been suggested in Japan. This study aims to better understand the potential undiagnosed ADHD population in Japan by quantifying the burden associated with ADHD symptoms through a comparison of the prevalence of comorbidities, health-related quality of life (HRQoL), work productivity and activity impairment (WPAI), and healthcare resource utilization (HRU) between undiagnosed potential ADHD respondents who were screened positive and negative using Adult ADHD Self-Report Scale (ASRS)-v1.1.

Methodology

Respondents from Japan National Health and Wellness Survey 2016 who answered ASRS-v1.1 without an ADHD diagnosis were included. Respondents checking ≥4 items from ASRS-A and ≥9 from ASRS-A+B were classified as ASRS A+ (n = 309) and ASRS AB+ (n = 227), respectively. ASRS negative (n = 9,280) were respondents who were neither ASRS A+ nor ASRS AB+. Data on the presence of comorbidities, HRQoL, WPAI, and HRU were compared.

Results

ASRS A+ and ASRS AB+ respondents reported higher coexistence of mental comorbidities (depression, generalized anxiety disorder, bipolar disorder, obsessive-compulsive disorder, etc.), sleep problems (insomnia, narcolepsy, sleep apnea, etc.), and physical comorbidities (non-alcoholic steatohepatitis, allergy, and asthma). They also reported greater WPAI and HRU and lower HRQoL than matched ASRS-negative respondents.

Conclusions

A significantly higher burden was identified among undiagnosed adults with potential ADHD symptoms. Appropriate diagnosis may help those at risk or those who present with symptoms overlapping with ADHD.

## Introduction

Attention-deficit/hyperactivity disorder (ADHD) is a common neuropsychiatric disorder that is characterized by inattention and/or excessive activity and impulsivity symptoms [1]. Symptoms of ADHD are usually observed in childhood and some symptoms, especially inattention rather than hyperactivity and impulsivity, can persist into adulthood [2]. These symptoms experienced by patients with ADHD frequently result in functional impairment across academic/occupational functioning, daily life, and social functioning [3].

ADHD prevalence was reported to be 6-10% in US children and adolescents between 1997 and 2016 [4]. In adults, the global prevalence of persistent ADHD and symptomatic ADHD was estimated at 2.58% and 6.76%, respectively [5]. Although the prevalence of ADHD in adults in the United States has been estimated to be 5.2% [6], the rates of diagnosed/treated patients have been reported as only 0.8% [7]. ADHD prevalence among Japanese adults was estimated to be around 1.65% [8], which is lower than the estimated 2.5% prevalence of ADHD in adults from a meta-analysis [9]. This could potentially indicate a sizable proportion of undiagnosed and consequently untreated ADHD patients in Japan.

Several studies have reported that diagnosed adult ADHD patients have high rates of comorbidities associated with psychiatric diseases, including depression and generalized anxiety disorder, and somatic diseases, including allergic diseases, obesity, and metabolic disorders, and are suffering from the disease burden of ADHD [10-13]. However, the burden of adults with ADHD symptoms without formal ADHD diagnosis has not been clarified yet.

ADHD in adults is diagnosed according to the guidelines of the Diagnostic and Statistical Manual of Mental Disorders, Fifth Edition (DSM-5) [1]. The Adult ADHD Self-Report Scale version 1.1 (ASRS-v1.1) Symptom Checklist is an extensively used 18-item screening tool that comprises 18 Category A symptoms from the DSM-Fourth Edition (DSM-IV) to identify potential ADHD adults and the severity of their symptoms [14,15]. The ASRS-v1.1 Symptom Checklist comprises a six-item Part A and a 12-item Part B [16]. Part A forms the basis for the ASRS-v1.1 Screener (ASRS-A) and has been validated as a pre-diagnostic screening tool to help recognize at-risk ADHD adults with a sensitivity of 91.4% and a specificity of 96.0% in the general population [14]. Part B provides additional information regarding the ADHD symptoms that respondents experienced [16]. Together, Part A and Part B form the full ASRS-v1.1 Symptom Checklist, which helps healthcare providers assess adult ADHD symptom profile and the level of impairment. The ASRS-v1.1 Symptom Checklist could also be categorized into the symptom domains of inattention and hyperactivity/impulsivity comprising nine items each, which is consistent with the DSM-IV ADHD criteria [15]. Updates based on the DSM-5 version of the screening scale of the ASRS-v1.1 instrument included the reduction of the minimum number of symptoms from six to five in either symptom domain (inattention or hyperactive/impulsive). This reduction in symptoms expanded the application of the screening scale to older adolescents and adults, allowing for more effective identification of adults with ADHD [17].

Although the ASRS-v1.1 instrument has been commonly used as an initial self-assessment tool and changes in the DSM-5 criteria increase the likelihood of identifying ADHD in adults, it remains unknown how the disease burden and baseline characteristics differ between respondents who had not been diagnosed with ADHD but were screened positive and negative. Knowing the potential disease burden and baseline characteristics of at-risk ADHD adults would help better inform and navigate them for proper diagnosis and treatment.

This study aims to address this by quantifying the burden associated with potential ADHD symptoms through a comparison of the prevalence of comorbidities, health-related quality of life (HRQoL), work productivity and activity impairment (WPAI), and healthcare resource utilization (HRU) between respondents screened ASRS-v1.1 positive and negative but not clinically diagnosed with ADHD in Japan.

## Materials and methods

Data source

The study utilized existing data from the Japan National Health and Wellness Survey (NHWS) conducted in 2016. The NHWS is a cross-sectional, internet-based, self-administered survey of a nationwide sample of respondents aged 18 or above. Respondents for the NHWS were recruited from the existing members of web-based opt-in consumer panels, and only those who provided informed consent were included in the study. Panel members were recruited through opt-in e-mails, e-newsletters, banner placements, and co-registration with panel partners. A stratified random sampling procedure, stratified by age and gender, was implemented to ensure that the demographic composition of the NHWS respondents was representative of the general adult population in Japan.

The NHWS survey was approved with exemption status upon review by Pearl Institutional Review Board (Indianapolis, IN, IRB Study Number: 16-KAN-124). All NHWS respondents provided informed consent prior to participating.

Study population

A random subset among all 2016 Japan NHWS respondents was selected and asked to complete the ASRS-v1.1 Symptom Checklist. Only respondents who responded to the ASRS-v1.1 Symptom Checklist were included in the study. Those who self-reported a diagnosis of ADHD by a physician were excluded. Respondents would indicate in the boxes of the copyrighted two-part ASRS-v1.1 Symptom Checklist that most closely represent the frequency of the occurrence for each symptom [15,16]. Responses for each question in each part of the ASRS-v1.1 Symptom Checklist were categorized into the number of darkly shaded boxes marked after the survey. The respondents were subsequently defined as the following: (1) “ASRS-A+” if at least four out of six questions were in the darkly shaded boxes in Part A; (2) “ASRS-AB+” if at least nine out of eighteen questions were in the darkly shaded boxes in Part A and Part B; and (3) “Control (ASRS-negative respondents)” if respondents were screened neither ASRS-A+ nor ASRS-AB+.

Measures, survey instruments, and outcome assessment

Demographic and general health characteristics measured in this study included gender, age, marital status, education, household income, employment status, Charlson Comorbidity Index (CCI) [18-20], body mass index (BMI), smoking status, alcohol use, and exercise behavior. Experience of ADHD-related symptoms was self-reported using the Japanese version of the ASRS-v1.1 Symptom Checklist [21].

Experience of comorbidities (anxiety, depression, generalized anxiety disorder, obsessive-compulsive disorder, panic disorder, phobias, post-traumatic stress disorder, social anxiety disorder, schizophrenia, bipolar disorder, insomnia, narcolepsy, sleep apnea, sleep difficulties, allergies, asthma, chronic obstructive pulmonary disease, high cholesterol, type 2 diabetes mellitus (T2DM), obesity, hypertension, alcoholism, and non-alcoholic steatohepatitis) was self-reported in the NHWS.

HRQoL was assessed using the Medical Outcomes Study Short-Form 36-Item Health Survey Version 2 (SF-36v2), an instrument used to measure an individual’s general health status [22]. The instrument is designed to report on eight health domains (physical functioning, role limitations due to physical problems, bodily pain, general health, vitality, social functioning, role limitations due to emotional problems, and mental health). The respondents completed this using the validated Japanese version of the SF-36v2 [23]. Scores from these domains can be used to calculate two summary scores: physical component summary (PCS) and mental component summary (MCS) scores. Summary scores were calculated using norm-based scoring algorithms. Higher scores indicate better quality of life. Health state utilities were evaluated using the EuroQol five dimension five-level (EQ-5D-5L) instrument that provides a simple, general measure of health through five questions [24]. The EQ-5D Index is a single number index value using a validated standard Japan value set [25]. An individual had better health status if they had higher scores.

The six-item WPAI validated questionnaire [26] was used to measure the impact of any health impairments on work and activities. In the WPAI, four measures of percentages (absenteeism, presenteeism, overall work productivity loss, and activity impairment) are generated, with higher percentages indicating more impairment due to health problems in the past seven days. Absenteeism measures percentage work time missed due to health, presenteeism measures the percentage of impairment at work due to health, overall work productivity loss measures impairment due to health using absenteeism and presenteeism measures, and activity impairment measures the percentage of impairment in daily activities due to health. Activity impairment measure is provided for all respondents but all other measures in the WPAI are only provided for respondents who reported work.

HRU was measured by the reported number of visits in the past six months to the emergency room (ER), healthcare providers (e.g., general and specialized physicians, internists, and dentists), and for hospitalizations because of the patient’s medical condition.

Analysis

To understand any underlying differences between respondents screened positive using ASRS-v1.1 Part A and controls, as well as between respondents screened positive using ASRS-v1.1 Part A+B and controls, a descriptive summary and comparison of their baseline demographic and health characteristics were made. Crude odds ratio (OR) of experiencing selected comorbidities among respondents screened positive using Part A and Part A+B compared to respondents screened negative were described.

A greedy matching algorithm was used to form the matched control groups for ASRS-A+ and ASRS-AB+ groups, respectively. Age, gender, marital status, education, employment status, household income, health insurance, CCI, BMI, alcohol consumption, and whether a respondent was a smoker were used in 1:4 propensity score matching. These factors were used in matching as previous studies have identified an association between the factors and health outcomes [27-29]. Bivariate comparisons were conducted after matching to ensure the covariates were balanced. Using 1:4 propensity score matching, the proportion of self-reported experience of comorbidities and health outcomes were compared between ASRS-v1.1-positive respondents and matched controls. Categorical variables and continuous variables were assessed using the chi-square test and one-way analyses of variance (ANOVA), respectively.

Statistical significance was evaluated at the 0.05 significance level during data analysis using SPSS Statistics Version 25 (IBM Corp., Armonk, NY, USA) [30] and R Version 3.4.4 [31].

## Results

Participants

**Figure 1:**
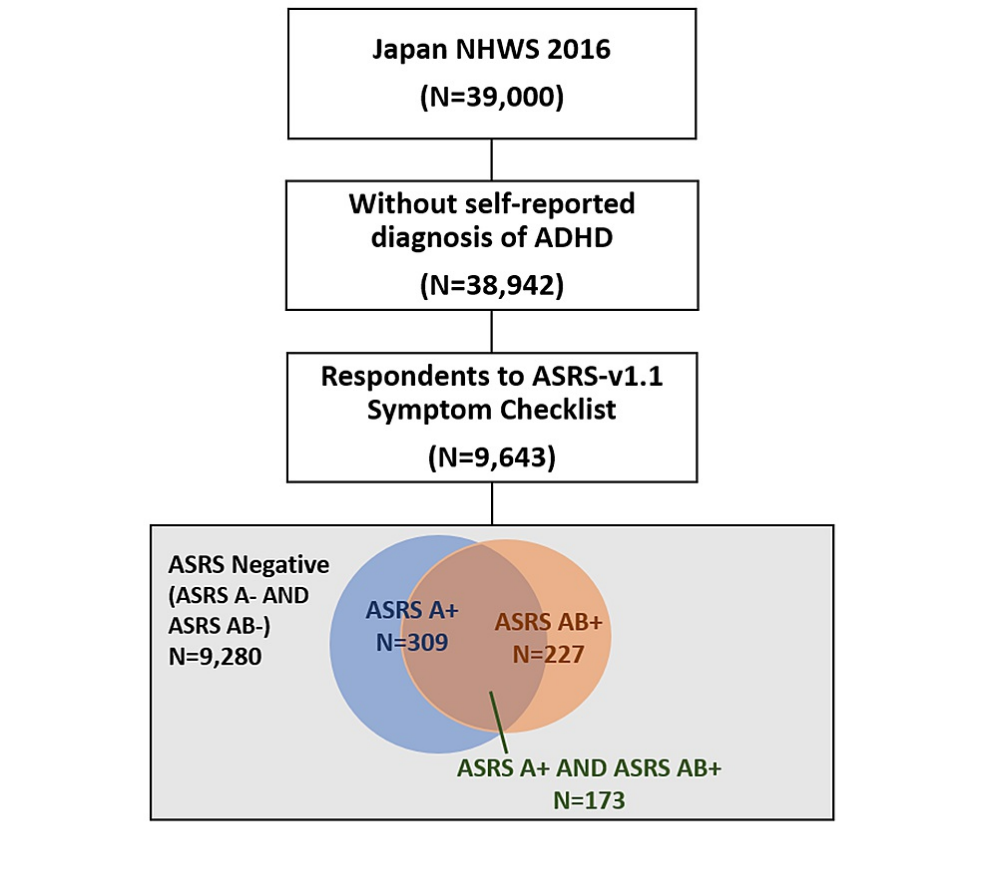
Respondent flow chart. Screened positive figures using Part A and using Part A+B are not exclusive. A total of 173 respondents were screened positive using both Part A and Part A+B. ASRS-negative are those who were screened negative for both Part A and Part A+B. ADHD: attention-deficit hyperactivity disorder; ASRS-v1.1: Adult ADHD Self-Report Scale version 1.1; NHWS: National Health and Wellness Survey

A total of 9,643 respondents without a diagnosis of ADHD who responded to the ASRS-v1.1 Symptom Checklist were included in the study. Of those, 9,280 (96.2% of all who responded to the ASRS-v1.1) were screened negative using Part A and Part A+B (control), 309 (3.2% of all who responded to ASRS-v1.1) were screened positive for Part A (ASRS-A+), and 227 (2.4%) were screened positive for Part A+B (ASRS-AB+). A total of 173 (1.8%) respondents were screened positive for both Part A and Part A+B (Figure 1). A total of 1,236 matched controls were identified for ASRS-A+ (matched ASRS-A-) and 908 matched controls for ASRS-AB+ (matched ASRS-AB-).

Demographic and general health characteristics

Compared to the respective ASRS-negative controls, ASRS-A+ and ASRS-AB+ respondents tended to be younger (mean [standard deviation: SD]: 42.91 [15.43] and 41.56 [15.23] vs. 50.74 [16.24]) and had a higher CCI (0.26 [0.80] and 0.23 [0.85] vs. 0.19 [0.56]) (Table 1). Compared to ASRS-negative controls, fewer ASRS-A+ and ASRS-AB+ respondents were married/living with partner (43.04% and 37.44% vs. 64.44%) and had completed university (39.48% and 40.09% vs. 45.04%). ASRS-A+ and ASRS-AB+ respondents also had lower household income compared to ASRS-negative respondents.

After matching, all demographic and general health characteristics were balanced between matched ASRS-A- and ASRS-A+ respondents, as well as between matched ASRS-AB- and ASRS-AB+ respondents (Table 4 in the Appendices).

**Table 1:** Demographic and general health characteristics among ASRS-negative, ASRS Part A positive (score ≥4) (ASRS-A+), and ASRS Part A+B positive (score ≥9) (ASRS-AB+) prior to matching. ASRS: Adult ADHD Self-Report Scale; BMI: body mass index; SD: standard deviation

		ASRS negative (N = 9,280)		ASRS-A+ (N = 309)		ASRS-AB+ (N = 227)	
Continuous variable		Mean	SD	Mean	SD	Mean	SD
Age		50.74	16.24	42.91	15.43	41.56	15.23
Charlson Comorbidity Index		0.19	0.56	0.26	0.80	0.23	0.85
ASRS score	ASRS Part A score	0.36	0.83	4.73	0.84	4.55	1.36
	ASRS Part B score	0.41	1.04	5.77	3.98	8.37	2.70
	ASRS Part A+B score	0.77	1.70	10.50	4.61	12.92	3.49
Categorical variable		Count	%	Count	%	Count	%
Gender	Male	4,888	52.67%	164	53.07%	115	50.66%
	Female	4,392	47.33%	145	46.93%	112	49.34%
Marital status	Married or living with partner	5,980	64.44%	133	43.04%	85	37.44%
	Divorced/separated/widowed	3,273	35.27%	171	55.34%	137	60.35%
	Decline to answer	27	0.29%	5	1.62%	5	2.20%
Level of education	Not completed university	5,014	54.03%	180	58.25%	129	56.83%
	Completed university education	4,180	45.04%	122	39.48%	91	40.09%
	Decline to answer	86	0.93%	7	2.27%	7	3.08%
Household income	Less than ¥3,000,000	1,509	16.26%	71	22.98%	57	25.11%
	¥3,000,000 to	2,246	24.20%	80	25.89%	50	22.03%
	¥5,000,000 to	2,259	24.34%	76	24.60%	48	21.15%
	¥8,000,000 or more	1,958	21.10%	46	14.89%	40	17.62%
	Decline to answer	1,308	14.09%	36	11.65%	32	14.10%
Employment status	Currently not employed	3,534	38.08%	122	39.48%	83	36.56%
	Currently employed	5,746	61.92%	187	60.52%	144	63.44%
BMI	Underweight (BMI < 18.5)	951	10.25%	28	9.06%	25	11.01%
	Normal weight (18.5 ≤ BMI ≤ 22.9)	4,590	49.46%	143	46.28%	101	44.49%
	Pre-obese (23 ≤ BMI ≤ 24.9)	1,615	17.40%	47	15.21%	32	14.10%
	Obese (BMI ≥ 25)	1,651	17.79%	71	22.98%	49	21.59%
	Decline to answer	473	5.10%	20	6.47%	20	8.81%
Smoking status	Current smoker	1,616	17.41%	51	16.50%	43	18.94%
	Former smoker	2,440	26.29%	77	24.92%	40	17.62%
	Never smoker	5,224	56.29%	181	58.58%	144	63.44%
Alcohol use	No use	3,092	33.32%	119	38.51%	95	41.85%
	Currently use alcohol	6,188	66.68%	190	61.49%	132	58.15%
Exercise status	No exercise	4,329	46.65%	169	54.69%	129	56.83%
	Currently exercise	4,951	53.35%	140	45.31%	98	43.17%

Prevalence of comorbidities among ASRS-positive and negative respondents

Prior to propensity score matching, significantly more ASRS-A+ and ASRS-AB+ respondents reported experiencing mental and neurological disorders, sleep problems, and physical illnesses (Table 2).

**Table 2:** Self-reported experience of comorbidities between unmatched ASRS-negative control group and ASRS-positive groups, between matched ASRS-A-negative and ASRS-A+ (score ≥4 for Part A), and between matched ASRS-AB-negative and ASRS-AB+ (score ≥9 for both Parts A and B). ^a^p < 0.05 comparing to ASRS-negative prior matching; ^b^p < 0.05 comparing to matched ASRS A-; ^c^p < 0.05 comparing to matched ASRS AB-. ASRS: Adult ADHD Self-Report Scale

	Prior matching		Matched ASRS-A- versus ASRS-A+				Matched ASRS-AB- versus ASRS-AB+			
	ASRS negative (N = 9,280)		Matched ASRS-A- (N = 1,236)		ASRS-A+ (N=309)		Matched ASRS-AB- (N = 908)		ASRS-AB+ (N = 227)	
Mental and neurological disorders	Count	%	Count	%	Count	%	Count	%	Count	%
Anxiety	1,328	14.31%	234	18.93%	119	38.51%^ab^	179	19.71%	80	35.24%^ac^
Depression	295	3.18%	53	4.29%	65	21.04%^ab^	38	4.19%	56	24.67%^ac^
Generalized anxiety disorder	33	0.36%	5	0.40%	15	4.85%^ab^	2	0.22%	16	7.05%^ac^
Obsessive-compulsive disorder	43	0.46%	12	0.97%	10	3.24%^ab^	6	0.66%	10	4.41%^ac^
Panic disorder	99	1.07%	16	1.29%	19	6.15%^ab^	18	1.98%	16	7.05%^ac^
Phobias	51	0.55%	14	1.13%	19	6.15%^ab^	11	1.21%	16	7.05%^ac^
Post-traumatic stress disorder	45	0.48%	7	0.57%	9	2.91%^ab^	6	0.66%	6	2.64%^ac^
Social anxiety disorder	97	1.05%	25	2.02%	36	11.65%^ab^	14	1.54%	27	11.89%^ac^
Schizophrenia	75	0.81%	17	1.38%	21	6.80%^ab^	11	1.21%	18	7.93%^ac^
Bipolar disorder	45	0.48%	10	0.81%	18	5.83%^ab^	4	0.44%	16	7.05%^ac^
Sleep problems	Count	%	Count	%	Count	%	Count	%	Count	%
Insomnia	637	6.86%	96	7.77%	87	28.16%^ab^	78	8.59%	63	27.75%^ac^
Narcolepsy	21	0.23%	4	0.32%	5	1.62%^ab^	6	0.66%	5	2.20%^ac^
Sleep apnea	249	2.68%	32	2.59%	24	7.77%^ab^	18	1.98%	12	5.29%^ac^
Sleep difficulties (other than insomnia, narcolepsy or sleep apnea)	99	1.07%	19	1.54%	16	5.18%^ab^	21	2.31%	16	7.05%^ac^
Physical conditions	Count	%	Count	%	Count	%	Count	%	Count	%
Allergies	951	10.25%	144	11.65%	58	18.77%^ab^	115	12.67%	51	22.47%^ac^
Asthma	297	3.20%	41	3.32%	26	8.41%^ab^	32	3.52%	23	10.13%^ac^
Chronic obstructive pulmonary disease	32	0.34%	4	0.32%	3	0.97%	2	0.22%	4	1.76%^ac^
High cholesterol	1,114	12.00%	113	9.14%	37	11.97%	69	7.60%	26	11.45%
Type 2 diabetes mellitus	379	4.08%	55	4.45%	14	4.53%	35	3.85%	13	5.73%
Obesity	1,651	17.79%	270	21.84%	71	22.98%^a^	198	21.81%	49	21.59%
Hypertension	1,370	14.76%	140	11.33%	36	11.65%	72	7.93%	28	12.33%^c^
Alcoholism	58	0.63%	1	0.08%	9	2.91%^ab^	5	0.55%	5	2.20%^ac^
Non-alcoholic steatohepatitis	43	0.46%	4	0.32%	6	1.94%^ab^	6	0.66%	5	2.20%^ac^

**Figure 2:**
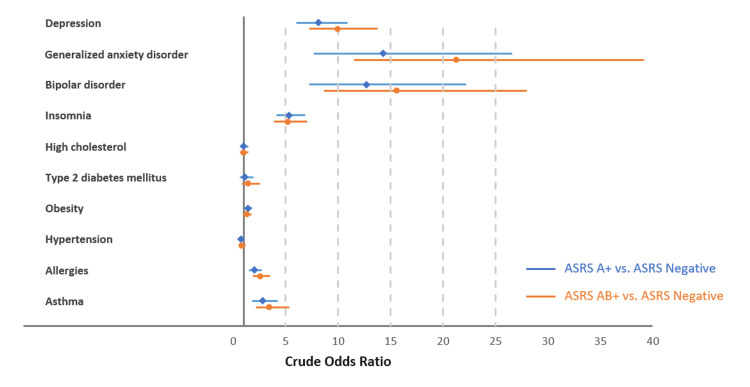
Crude odds ratio of experiencing selected comorbidities among respondents screened positive using ASRS-v1.1 compared to respondents screened negative. Error bar represents 95% confidence interval for the crude odds ratio. ASRS-v1.1: Adult ADHD Self-Report Scale version 1.1

From an association perspective, ASRS-A+ and ASRS-AB+ respondents were significantly more likely to experience depression (OR [95% confidence interval: CI]: 8.11 [6.03, 10.92] and 9.97 [7.22, 13.78]), generalized anxiety disorder (14.30 [7.68, 26.61] and 21.25 [11.52, 39.20]), bipolar disorder (12.69 [7.26, 22.20] and 15.56 [8.66, 27.98]), insomnia (5.32 [4.10, 6.90] and 5.21 [3.86, 7.05]), allergies (2.02 [1.51, 2.71] and 2.54 [1.85, 3.49]), and asthma (2.78 [1.83, 4.22] and 3.41 [2.18, 5.33]) than ASRS-negative controls (Figure 2).

After propensity score matching, a significantly higher proportion of ASRS-A+ respondents experienced mental and neurological disorders, including anxiety, depression, generalized anxiety disorder, obsessive-compulsive disorder, panic disorder, phobias, post-traumatic stress disorder, social anxiety disorder, schizophrenia, and bipolar disorder, compared to matched ASRS-A- respondents. Significantly more ASRS-A+ respondents also experienced sleep problems, including insomnia, narcolepsy, sleep apnea, and other sleep difficulties (other than insomnia, narcolepsy, or sleep apnea), compared to matched ASRS-A- respondents. In addition, significantly more ASRS-A+ respondents also reported experiencing some physical illnesses, including allergies, asthma, alcoholism, and non-alcoholic steatohepatitis, compared to matched ASRS-A- respondents (Table 2). Similar to ASRS-A+ respondents, significantly more respondents screened ASRS-AB+ also reported experiencing similar mental and neurological disorders, sleep problems, and physical illnesses compared to matched ASRS-AB- respondents (Table 2).

Health outcomes assessment among ASRS-positive and negative respondents

After propensity score matching, ASRS-A+ respondents reported significantly lower HRQoL relative to matched ASRS-A- respondents in PCS (48.02 [8.27] vs. 52.72 [6.55], p < 0.001), MCS (34.44 [11.69] vs. 45.90 [9.66], p < 0.001), and EQ-5D-5L index (0.68 [0.21] vs. 0.84 [0.16], p < 0.001) (Table 3). ASRS-A+ respondents reported significantly increased absenteeism (12.01% [23.32%] vs. 4.10% [14.99%], p < 0.001), presenteeism (46.42% [29.77%] vs. 20.94% [24.85%], p < 0.001), total work productivity impairment (49.82% [30.37%] vs. 22.60% [26.24%], p < 0.001), and total activity impairment (48.64% [28.88%] vs. 23.34% [25.56%], p < 0.001) compared to matched ASRS A- respondents. The impairment was more than two times that of the matched ASRS-A- respondents. In terms of HRU, compared to matched ASRS A- respondents, ASRS A+ respondents had significantly more visits to the HCP (7.85 [11.83] vs. 4.59 [7.40], p < 0.001) and reported more numbers of hospitalizations (2.16 [15.03] vs. 0.82 [6.79], p = 0.020) in the past six months (Table 3).

**Table 3:** HRQoL, WPAI, and HRU between matched ASRS-negative and ASRS-A+ (score ≥4 for Part A) and between matched ASRS-negative and ASRS-AB+ (score ≥9 for both Parts A and B). ASRS: Adult ADHD Self-Report Scale; EQ-5D-5L: ER: emergency room; EuroQol five dimension five-level; HCP: healthcare provider; HRU: healthcare resource utilization; SD: standard deviation; WPAI: work productivity and activity impairment

	Matched ASRS-A- versus ASRS-A+					Matched ASRS-AB- versus ASRS-AB+				
	Matched ASRS A- (N = 1,236)		ASRS A+ (N = 309)		P-value	Matched ASRS AB- (N = 908)		ASRS AB+ (N = 227)		P-value
Health-related quality of life	Mean	SD	Mean	SD		Mean	SD	Mean	SD	
Physical component summary	52.72	6.55	48.02	8.27	<0.001	52.94	6.29	47.42	8.22	<0.001
Mental component summary	45.90	9.66	34.44	11.69	<0.001	45.82	9.66	32.60	11.31	<0.001
EQ-5D-5L index	0.84	0.16	0.68	0.21	<0.001	0.85	0.16	0.66	0.21	<0.001
Work productivity and activity impairment	Mean	SD	Mean	SD	P-value	Mean	SD	Mean	SD	P-value
Absenteeism	4.10%	14.99%	12.01%	23.32%	<0.001	3.57%	12.59%	15.14%	24.37%	<0.001
Presenteeism	20.94%	24.85%	46.42%	29.77%	<0.001	22.07%	24.49%	51.25%	29.15%	<0.001
Total work productivity impairment	22.60%	26.24%	49.82%	30.37%	<0.001	23.99%	25.86%	55.84%	29.16%	<0.001
Total activity impairment	23.34%	25.56%	48.64%	28.88%	<0.001	23.99%	25.83%	53.13%	27.56%	<0.001
Healthcare resource utilization	Mean	SD	Mean	SD	P-value	Mean	SD	Mean	SD	P-value
Number of HCP visits in past 6 months	4.59	7.40	7.85	11.83	<0.001	3.99	6.90	8.03	11.52	<0.001
Number of ER visits in the past 6 months	0.08	0.62	0.37	5.13	0.056	0.04	0.31	0.48	5.98	0.029
Number of hospitalizations in the past 6 months	0.82	6.79	2.16	15.03	0.020	0.60	5.00	2.41	17.15	0.006

Similarly, after propensity score matching, ASRS-AB+ respondents reported significantly lower HRQoL relative to matched ASRS-AB- respondents in PCS (47.42 [8.22] vs. 52.94 [6.29], p < 0.001), MCS (32.60 [11.31] vs. 45.82 [9.66], p < 0.001), and EQ-5D-5L index (0.66 [0.21] vs. 0.85 [0.16], p < 0.001) (Table 3). ASRS AB+ respondents reported significantly increased absenteeism (15.14% [24.37%] vs. 3.57% [12.59%], p < 0.001), presenteeism (51.25% [29.15%] vs. 22.07% [24.49%], p < 0.001), work productivity impairment (55.84% [29.16%] vs. 23.99% [25.86%], p < 0.001), and activity impairment (53.13% [27.56%] vs. 23.99% [25.83%], p < 0.001) compared to matched ASRS-AB- respondents. The impairment was also more than two times that of the matched ASRS-AB- respondents. In terms of HRU, compared to matched ASRS-AB- respondents, ASRS-AB+ respondents had significantly more HCP visits (8.03 [11.52] vs. 3.99 [6.90], p < 0.001) and ER visits (0.48 [5.98] vs. 0.04 [0.31], p = 0.029) and reported more hospitalizations (2.41 [17.15] vs. 0.60 [5.00], p = 0.006) in the past six months (Table 3).

## Discussion

In this study, we investigated the physical and mental burden among the population in Japan who remained undiagnosed with ADHD but were screened to have potential ADHD symptoms using the ASRS-v1.1 Screener and Symptom Checklist. Among the 9,643 respondents who were undiagnosed with ADHD, 3.2% (n = 309) and 2.4% (n = 227) were found to have been screened ASRS-A+ and ASRS-AB+, respectively. Only 1.8% (n = 173) were screened positive for both ASRS-A and ASRS-AB. In Japan, the prevalence of diagnosed ADHD was previously reported to be 1.65% [8], which is lower than the 2.5-3.4% average prevalence among adults worldwide [6,9]. If we consider our study population, that is, those who were undiagnosed with ADHD but were screened positive using the ASRS-v1.1 instrument, the prevalence of ADHD in Japan may be higher and closer to the current worldwide prevalence.

In our study, those who were undiagnosed for ADHD but were screened ASRS-positive (irrespective of ASRS-A or ASRS-AB) tended to be younger, had lower income levels, and were significantly less likely to be married or living with a partner or have completed a university education compared to non-ADHD controls (Table 1). The sociodemographic differences in income levels and marital status observed between the two groups could have been because the ASRS-A+ and ASRS-AB+ groups were significantly younger than the matched control group. This is also consistent with previously published findings demonstrating poorer academic, social, and occupational functioning among adults diagnosed with ADHD [3,12].

In terms of health characteristics, previous studies have reported a higher risk of substance use and dependence, for example, consumption of alcohol and smoking, in patients with ADHD [32,33]. In addition, a possible genetic basis for the co-occurrence of substance use disorder and ADHD has been reported [34]. Our study findings supported this, though fewer ASRS+ respondents reported currently drinking alcohol, alcoholism occurred at an approximately 4-36-fold greater rate among ASRS-positive respondents compared to ASRS-negative respondents.

Our study showed that ASRS-positive respondents with potential ADHD symptoms experienced significantly more mental and neurological comorbidities (e.g., depression, generalized anxiety disorder, bipolar disorder, insomnia, social anxiety disorder) and physical comorbidities (e.g., allergies, asthma, sleep apnea, non-alcoholic steatohepatitis), even after propensity score matching. This is in line with previous studies reporting that ADHD patients have higher rates of other comorbid mental disorders with symptoms overlapping ADHD [12,35]. Compared to the study by Kirino et al., our study found a smaller difference in the proportion of respondents with comorbid mental disorders compared to matched controls, that is, depression, generalized anxiety disorder, and sleep difficulties. This may be attributed to the possibility that other comorbid conditions of the undiagnosed ADHD population also remain undiagnosed compared to diagnosed patients. This also highlights the importance of appropriate diagnosis of ADHD and other comorbidities.

While Kooij et al. only focused on central nervous system (CNS)-related conditions, other studies have investigated the association between ADHD and physical disorders such as inflammation, allergic diseases [13,36], obesity, T2DM, and hypertension [10,37]. For example, Wang et al. reported a significant association between ADHD risk and the presence of rhinitis and eczema symptoms among children [36]. Likewise, our study showed that allergies were approximately two-fold more common compared to matched controls. Although causal mechanisms remain unknown, this may further support the theory of a shared etiology, linking an increase in proinflammatory biochemical mechanisms to physical comorbidities as well as brain circuits associated with emotional and behavior control in patients with ADHD [13]. These psychiatric symptoms have been reported to present more severely with the co-occurrence of ADHD and other psychiatric disorders [38]. It has been reported that adults with ADHD also have a higher prevalence of T2DM, obesity, and hypertension compared to those without ADHD [10,37]. Although a significant association between ADHD and obesity has been reported [39], a higher prevalence of obesity was not evident among adults with ADHD symptoms in this study. This might be due to the lower rate and/or milder level of obesity in Japan compared to Western populations [40], or due to the adjustment of BMI used in matching. It could be that obesity might be lower in undiagnosed adults with ADHD symptoms compared to diagnosed ADHD patients. In our study, the prevalence for T2DM and hypertension among those with ADHD symptoms were 4.5-5.7% and 11.7-12.3% respectively, compared to 3.9% and 8.5%, respectively, in adults with ADHD from previous reports [37]. Matched ASRS-negative controls in our study also had a higher prevalence of 3.9-4.4% for T2DM and 7.9-11.3% for hypertension compared to 1.6% and 4.5%, respectively, for adults without ADHD in previous reports [37]. While our analysis showed there were no statistically significant differences in T2DM and hypertension between ASRS-positive and negative groups, it is possible that the higher prevalence of somatic comorbidities and other differences observed between ASRS-positive and negative groups in our study was due to the higher mean age of the control group.

It is well established that ADHD with or without the presence of comorbidities contributes to poorer HRQoL [11,12]. Our study findings showed that this was true even among those undiagnosed for ADHD but were screened ASRS-positive. After propensity score matching, our study showed that the presence of ADHD symptoms itself was significantly associated with poorer HRQoL, not just in the MCS, but also poorer physical component scores. As for EQ-5D-5L, it has been reported as 0.74 among diagnosed adults with ADHD [11], and it was even lower (0.59) among those with psychiatric comorbidities [41]. In our study, EQ-5D-5L of 0.66 and 0.68 among undiagnosed adults with ADHD symptoms fell within the range above and was significantly lower than ASRS-negative respondents (0.84 and 0.85). However, how the EQ-5D-5L score is associated with comorbidities in undiagnosed adults with ADHD still needs to be investigated.

While our study could not evaluate the severity of ADHD symptoms, the ASRS+ group had more health problems, as shown by their significantly greater healthcare resource use compared to controls. The ASRS+ group reported approximately 1.7-2.0 times more physician visits, 4.6-12.0 times more emergency room visits, and 2.6-4.0 times more hospitalizations than their respective matched controls (Table 3). Because the study participants of this study have never been diagnosed and treated for ADHD, it is assumed that HRU was due to comorbidities other than ADHD or due to their increased risk of injuries [42]. Moreover, despite treatments for comorbidities, lower QoL and impaired work productivity were observed in undiagnosed adults with ADHD symptoms.

Similar to what has been reported for patients with ADHD [12], poorer occupational and social functioning was observed in ASRS+ respondents. The mean overall WPAI scores for the ASRS+ groups were over two times higher compared to the control group. Compared to matched controls, they reported a greater degree of absenteeism and presenteeism and impaired ability to engage in general daily activities due to health problems. It is known that overall work impairment was high (45.65%) in undiagnosed adults with ADHD symptoms [11]. It has also been reported that overall work impairment was higher (60.8%) in diagnosed ADHD patients with comorbidities [41]. In this study which included respondents with various comorbidities, overall work impairment was 49.82-55.84%. This was as severe as that reported for diagnosed ADHD patients with/without comorbidities. Absenteeism and presenteeism for major depressive disorder (MDD) patients in Canada were also reported as 22.2% and 61.5%, respectively [43]. As for MDD patients in Japan, absenteeism and presenteeism have been reported as 14.8% and 38.0%, respectively [44], contributing to significant disability and socioeconomic burden in Japan. Although some respondents with comorbid MDD were included in this study, the results showed that absenteeism (12.0-15.1%) and presenteeism (46.4-51.3%) were sizable in undiagnosed adults with ADHD symptoms. Moreover, having ADHD symptoms has been associated with similar impairment reported previously among patients with MDD.

While most studies highlighted the burden of ADHD among diagnosed adults compared to adults without ADHD, there remains a paucity of information regarding the potential burden associated with missed ADHD symptoms among the undiagnosed population. A cross-sectional, retrospective analysis of 247 patients revealed that the presence of any psychiatric symptom, in the presence or absence of a formal diagnosis, had a negative impact on the patient’s quality of life [45]. Here, the results from the study revealed that adults with potential ADHD symptoms experienced significant humanistic burden, although undiagnosed. It has been established that treatment for ADHD improves long-term outcomes in ADHD patients, which would remain poor otherwise [46]. A future consideration is to evaluate how proper diagnosis and appropriate treatment for ASRS-positive individuals may improve their outcomes.

Limitations

Due to the cross-sectional nature of the NHWS data, no causal inference can be made. As the data were provided by self-administered questionnaires, it is subject to recall bias, and no verification was done for patient-reported outcomes. While the NHWS sample was made to be predominantly representative of the adult population in Japan, the representativeness of ASRS+ respondents to the larger population is uncertain. As the NWHS was an online self-administered questionnaire, respondents with Internet access and those better-versed in technology might be relatively well-educated or have high socioeconomic status compared to those without access to this survey. Therefore, generalizability might be considered. Our study did not evaluate the severity of ADHD symptoms nor did it explore the potential presentation type of ADHD symptoms (i.e., inattention or hyperactive/impulsive or combined); therefore, no comparison can be made with patients diagnosed with ADHD nor any association of the observed burden among ASRS+ respondents with the ADHD presentation type. Although ASRS is a screener of ADHD, it is possible that symptoms of comorbidities can increase the likelihood of screening ASRS positive. Thus, it is worth noting that ASRS-positive individuals may not necessarily be clinically diagnosed with ADHD. Our study did not exclude the possibility of the involvement of comorbidities in ADHD symptoms.

## Conclusions

Undiagnosed adults who demonstrate potential ADHD symptoms in Japan experience a significant personal and professional burden. They reported a substantial prevalence of comorbidities and suffer decreased QoL, impaired work productivity, and greater HRU, similar to patients diagnosed with ADHD, compared to the ASRS-negative population. These preliminary results raise considerations to take note of to ensure the appropriate diagnosis of those at risk for ADHD or for those who present with symptoms overlapping with ADHD. Future studies that assess comorbidities, HRQOL, WPAI, and HRU focusing specifically on clinically diagnosed ADHD patients could provide further insights into the disease burden of ADHD in Japan.

## Appendices

**Table 4:** Demographic and general health characteristics between matched ASRS-negative and ASRS Part A positive (score ≥4) (ASRS-A+) and between matched ASRS-negative and ASRS Part A+B positive (score ≥9) (ASRS-AB+). ASRS: Adult ADHD Self-Report Scale; BMI: body mass index; SD: standard deviation

		Matched ASRS-A- versus ASRS-A+						Matched ASRS-AB- versus ASRS-AB+					
		Matched ASRS-A- (N = 1,236)		ASRS-A+ (N = 309)		P-value	Standardized mean difference	Matched ASRS-AB- (N = 908)		ASRS-AB+ (N = 227)		P-value	Standardized mean difference
Continuous variable		Mean	SD	Mean	SD			Mean	SD	Mean	SD		
Age		41.92	16.19	42.91	15.43	0.332	0.063	41.55	15.69	41.56	15.23	0.992	0.001
Charlson comorbidity index		0.24	0.73	0.26	0.80	0.681	0.025	0.19	0.62	0.23	0.85	0.377	0.059
Categorical variable		Count	%	Count	%	P-value	Standardized mean difference	Count	%	Count	%	P-value	Standardized mean difference
Gender	Male	639	51.70%	164	53.07%	0.665	0.028	481	52.97%	115	50.66%	0.533	0.046
	Female	597	48.30%	145	46.93%			427	47.03%	112	49.34%		
Marital status	Married or living with partner	522	42.23%	133	43.04%	0.867	0.033	327	36.01%	85	37.44%	0.549	0.076
	Divorced/separated/widowed	698	56.47%	171	55.34%			569	62.67%	137	60.35%		
	Decline to answer	16	1.29%	5	1.62%			12	1.32%	5	2.20%		
Level of education	Not completed university	727	58.82%	180	58.25%	0.738	0.047	507	55.84%	129	56.83%	0.955	0.023
	Completed university education	489	39.56%	122	39.48%			374	41.19%	91	40.09%		
	Decline to answer	20	1.62%	7	2.27%			27	2.97%	7	3.08%		
Household income	Less than ¥3,000,000	275	22.25%	71	22.98%	0.972	0.046	233	25.66%	57	25.11%	0.954	0.061
	¥3,000,000 to	308	24.92%	80	25.89%			193	21.26%	50	22.03%		
	¥5,000,000 to	320	25.89%	76	24.60%			181	19.93%	48	21.15%		
	¥8,000,000 or more	178	14.40%	46	14.89%			179	19.71%	40	17.62%		
	Decline to answer	155	12.54%	36	11.65%			122	13.44%	32	14.10%		
Employment status	Currently not employed	488	39.48%	122	39.48%	1.000	<0.001	322	35.46%	83	36.56%	0.757	0.023
	Currently employed	748	60.52%	187	60.52%			586	64.54%	144	63.44%		
BMI	Underweight (BMI < 18.5)	124	10.03%	28	9.06%	0.952	0.053	92	10.13%	25	11.01%	0.960	0.059
	Normal weight (18.5 ≤ BMI ≤ 22.9)	590	47.73%	143	46.28%			425	46.81%	101	44.49%		
	Pre-obese (23 ≤ BMI ≤ 24.9)	176	14.24%	47	15.21%			122	13.44%	32	14.10%		
	Obese (BMI ≥ 25)	270	21.84%	71	22.98%			198	21.81%	49	21.59%		
	Decline to answer	76	6.15%	20	6.47%			71	7.82%	20	8.81%		
Smoking status	Current smoker	198	16.02%	51	16.50%	0.974	0.015	174	19.16%	43	18.94%	0.924	0.029
	Former smoker	313	25.32%	77	24.92%			150	16.52%	40	17.62%		
	Never smoker	725	58.66%	181	58.58%			584	64.32%	144	63.44%		
Alcohol use	No use	466	37.70%	119	38.51%	0.793	0.017	379	41.74%	95	41.85%	0.976	0.002
	Currently use alcohol	770	62.30%	190	61.49%			529	58.26%	132	58.15%		
Exercise status	No exercise	667	53.96%	169	54.69%	0.818	0.015	534	58.81%	129	56.83%	0.588	0.040
	Currently exercise	569	46.04%	140	45.31%			374	41.19%	98	43.17%		
